# Palmitoleate protects against lipopolysaccharide-induced inflammation and inflammasome activity

**DOI:** 10.1016/j.jlr.2024.100672

**Published:** 2024-10-11

**Authors:** Prakash Kumar Sahoo, Aiswariya Ravi, Baolong Liu, Jiujiu Yu, Sathish Kumar Natarajan

**Affiliations:** 1 Department of Nutrition & Health Sciences, University of Nebraska-Lincoln, Lincoln, NE, USA; 2 Shaanxi Key Laboratory of Molecular Biology for Agriculture, College of Animal Science and Technology, Northwest A&F University, Yang ling, Shaanxi, China; 3 Department of Nutrition, Case Western Reserve University, Cleveland, OH, USA; 4 College of Allied Health Professions Medical Nutrition Education, University of Nebraska Medical Center, Omaha, NE, USA; 5 Department of Biochemistry, University of Nebraska-Lincoln, Lincoln, NE, USA

**Keywords:** monounsaturated fatty acids, macrophages, trophoblasts, placenta, obesity, mitogen-activated protein kinase, pregnancy

## Abstract

Inflammation is part of natural immune defense mechanism against any form of infection or injury. However, prolonged inflammation could perturb cell homeostasis and contribute to the development of metabolic and inflammatory diseases, including maternal obesity, diabetes, cardiovascular diseases, and metabolic dysfunction–associated steatotic liver diseases (MASLD). Polyunsaturated fatty acids have been shown to mitigate inflammatory response by generating specialized proresolving lipid mediators, which take part in resolution of inflammation. Similarly here, we show that palmitoleate, an omega-7 monounsaturated fatty acid exerts anti-inflammatory properties in response to lipopolysaccharide (LPS)-mediated inflammation. Exposure of bone marrow–derived macrophages (BMDMs) to LPS or TNFα induces robust increase in the expression of proinflammatory cytokines and supplementation of palmitoleate inhibited LPS-mediated upregulation of proinflammatory cytokines. We also observed that palmitoleate was able to block LPS + ATP-induced inflammasome activation mediated cleavage of procaspase 1 and prointerleukin-1β. Further, treatment of palmitoleate protects against LPS-induced inflammation in human THP-1–derived macrophages and trophoblasts. Coexposure of LPS and palmitate (saturated free fatty acid) induces inflammasome and cell death in BMDMs, however, treatment of palmitoleate blocked LPS and palmitate-induced cell death in BMDMs. Further, LPS and palmitate together results in the activation of mitogen-activated protein kinases and pretreatment of palmitoleate inhibited the activation of mitogen-activated protein kinases and nuclear translocation of nuclear factor kappa B in BMDMs. In conclusion, palmitoleate shows anti-inflammatory properties against LPS-induced inflammation and LPS + palmitate/ATP-induced inflammasome activity and cell death.

## Graphical abstract

**Figure:**
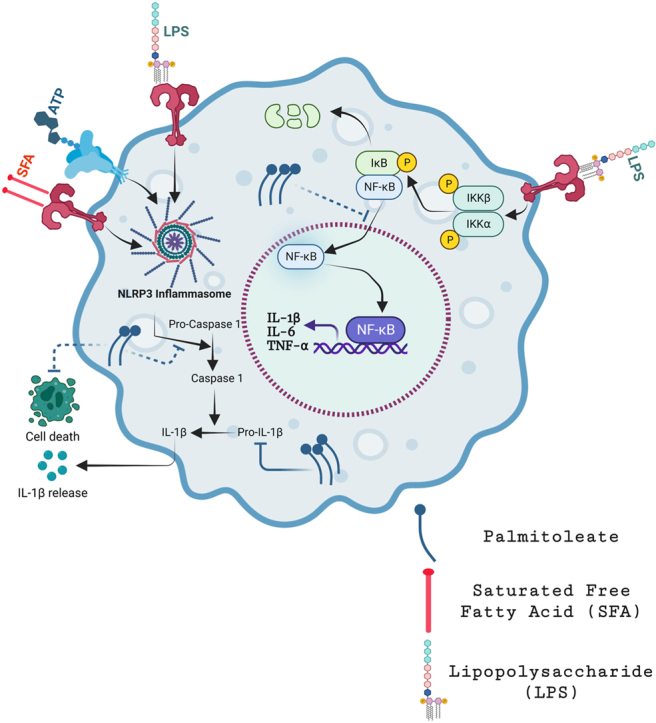

Inflammation is a normal physiological cellular response to any form of injury or infection, resulting in the clearance of the damaged tissue and infected cells (1). Inflammation has been commonly viewed as a harmful phenomenon, and is associated with the disruption of cell and tissue homeostasis (2). Inflammation is particularly helpful in sensing danger and mitigating the danger signals, however chronic uncontrolled inflammation has the potential to cause severe damage to tissues, perturbing tissue homeostasis and contribute to inflammatory diseases (3). Inflammation-mediated cellular stress also has the potential to activate innate immune responses including inflammasome (4). Inflammasomes are cytosolic multiprotein complexes that assemble in a response to pathogen-associated molecular patterns (PAMPs) and danger-associated molecular patterns (DAMPs). These PAMPs or DAMPs are recognized by pathogen recognition receptors and further regulate the expression of interferons and proinflammatory cytokines (3).

Maternal obesity (MO) is associated with the activation of systemic inflammation. Proinflammatory cytokines like TNF-α, C-reactive protein, and CD14, a coreceptor of toll-like receptor-4 (TLR4) were reported to be elevated in maternal circulation with obesity (5, 6, 7, 8, 9). In addition, MO-induced increased levels of TNF-α, interleukin (IL)-1β, IL-6, IL-8, monocyte chemoattractant protein-1, and C-X-C motif chemokine receptor 2 in the placenta were reported (6, 7, 9). Activation of c-Jun N-terminal kinase (JNK), a mitogen-activated protein kinase (MAPK), nuclear factor kappa B (NF-kB), and signal transducer and activator of transcription-3 were reported to be activated in the placenta during MO, further supporting mechanistic insights in the activation of proinflammatory signaling processes (6, 10).

Increased placental inflammation in MO is related to the increased FFAs and the TLR4-induced proinflammatory signaling pathway (6). Further, an obesogenic environment with elevated FFAs and glucose in the fetal circulation activates TLR4-dependent pathways to promote inflammation in the offspring during MO (11, 12, 13, 14). MO induces metabolic endotoxemia and inflammation to the placenta, maternal adipose tissue, and circulating macrophage, respectively, thus suggesting metabolic inflammation or metainflammation (15, 16, 17). Further, MO also increased recruitment of neutrophils into the placenta to cause oxidative stress and inflammation (18, 19). Placental inflammation increases the expression of fatty acid transport protein (FATP) and amino acids transport in the placenta and insulin resistance to pregnant mothers with obesity (20). Increased FATP2 levels were observed in the placental basolateral plasma membrane from obese mothers and showed increased correlation with increasing maternal BMI (21). High maternal BMI also increased the expression of FATP6 and fatty acid translocase in the placenta of obese mothers compared to control (22). Increased FATP in basolateral membrane suggests increased transport of fatty acids to offspring during MO, resulting in the development of macrosomia (23, 24).

As part of the innate immune function of cells, inflammasome activation is central to recognition, subsequent initiation of cellular responses, and clearance of the noxious agents (25). While appropriate inflammasome activation is crucial in mounting an effective response to foreign antigen presence; aberrant and uncontrolled inflammasome activation has been implicated in various inflammatory pathologies, including autoimmune diseases and metabolic syndrome (26). NACHT, LRR, and PYD domain–containing protein 3 (NLRP3) inflammasome is the most widely studied inflammasome and has been recognized as a key mediator in the pathogenesis of diverse inflammatory syndromes (27). NLRP3 inflammasome assembly is a two-step process; an initial priming of cells by cytokines or PAMPs and a secondary activation signal (particulates, crystals, and ATP) culminating with active caspase-1–dependent secretion of proinflammatory IL-1β and IL-18 (28). Further, saturated FFAs (palmitate [PA] and stearate) and oxidized lipids have also been shown to prime and activate NLRP3 inflammasome assembly, predominantly observed during metabolic disease development (3, 29, 30).

*Palmitoleate (PO), an omega-7 MUFA*, has been shown to act as a lipokine and an anti-inflammatory lipid mediator during obesity and metabolic diseases. Biosynthesis of PO in the trophoblasts was decreased in MO compared to the trophoblasts isolated from healthy placenta (31). Further, circulating levels of PO in mothers and umbilical vein of fetus was decreased with MO. Placental PO was also decreased in both male and female placentas of obese mothers, and oleate was only decreased in male placentas. Decreased PO was correlated with the development of maternal insulin resistance and metainflammation in the maternal systemic circulation, placenta, and fetus, respectively, which leads to our investigation on the protective role of PO against NLRP3 inflammasome and its priming and activation (31). We hypothesize that PO protects against inflammation and inflammasome activity in BMDMs and placental trophoblasts.

## Materials and methods

### Materials

All chemicals and buffers were of analytical grade and purchased from Thermo Fisher Scientific (Massachusetts). PA (A3803), PO (P9417), LPS (L2630), and fatty acid–free BSA (P5585) were obtained from MilliporeSigma.

### Antibodies and other reagents

Primary antibodies against phospho (p)-JNK (# 9251), JNK (# 9252), p-ERK half (# 9109), ERK 1/2 (# 4695), p-p38 (# 9211), p38 (# 9212), histone deacetylase 1 (# 34589), NLRP3 (# 15101), caspase 1 (# 3866), cleaved caspase 1 (# 4199), IL-1β (# 12703), cleaved IL-1β (# 83186), and NF-κB pathway antibody sampler kit (# 64662) were purchased from Cell Signaling Technologies (Danvers, MA). Actin antibody (# A-5441) was from MilliporeSigma (Burlington, MA). Mouse TNFα (#ab259411) from Abcam (Waltham, MA). Peroxidase-conjugated secondary antibodies were obtained from Jackson ImmunoResearch lab (West Grove, PA).

### Cell culture

HTR-8/SVneo (HTR-8) (CRL-3271), normal human immortalized first trimester placental trophoblast cells, choriocarcinoma-derived third trimester placental trophoblast cell line JEG-3 (HTB-36), and mouse macrophage cell line, RAW 264.7 (TIB-71) were obtained from ATCC and used. HTR-8 and RAW 264.7 cells were maintained in DMEM (Corning) supplemented with 10% FBS (Gibco) and 0.01% plasmocin. JEG-3 cells were maintained in MEM (Corning) supplemented with 10% FBS (Gibco) and 0.01% plasmocin to avoid mycoplasma contamination. Cells were maintained at 37°C in a 5% CO2-humidified incubator and passaged regularly (usually 3–4 days). THP-1 cells were cultured in RPMI-1640 (ATCC) supplemented with 10% FBS and 0.05 mM 2-mercaptoethanol. THP-1 cells were passaged every 3–4 days and maintained until 10 passages.

### Differentiation of human leukemia monocytic cell line (THP-1) to macrophage-like phenotype

THP-1 cells are monocytic in nature and can be differentiated to macrophage-like phenotype using phorbol 12-myristate 13-acetate (PMA, MilliporeSigma). Briefly, cells were collected using centrifugation and resuspended appropriately. Treatment of PMA (150 nM) for 2 days induces differentiation into THP-1–derived macrophages (THP-1 DMs). After 2 days, the cells were washed twice with complete medium (without PMA) and maintained for additional 2 days to obtain resting macrophage like cells.

### Bone marrow isolation from mice and differentiation to macrophage

Mouse BMDMs were cultured as described (32). Briefly, primary bone marrow cells were collected from the femur and tibia bones of C57BL/6J mice and cultured in RPMI 1640 medium (Corning, Tewksbury, MA) containing 10% FBS (Atlanta Biologicals, Minneapolis, MN, S1150), 25% L929 cell-conditioned medium, 50 μg/ml Pen-Strep (Corning), 2 mM glutamine (Corning), 1 mM sodium pyruvate (Corning), and 10 mM Hepes buffer (Corning). These primary cells were cultured at 37°C in 5% CO2 for 6–8 days until macrophages reached 80–90% confluency.

### *In vitro* induction of inflammation and inflammasome using LPS, TNFα or LPS, and ATP/PA

To induce inflammation in vitro, we used LPS or TNFα at a concentration of 10 ng/ml for BMDM and THP-1 differentiated macrophages and 1 μg/ml for trophoblasts. Macrophages were exposed to TNFα for 30 min or LPS for 3 h and trophoblasts were exposed with to LPS for 4 h. To induce inflammasome activation *in vitro*, cells were exposed with LPS for 3/4 h for macrophages and trophoblasts, respectively, followed by treatment with either ATP (30 min, 5 mM) or PA (16 h, 200–400 μM) for inflammasome activation. Cells were scrapped and used to isolate mRNA Trizol reagent or cell lysate preparations.

### Fatty acid preparation and cell treatment

Fatty acids (PA or PO) were prepared by dissolving PA and PO in isopropanol to a stock concentration of 80 mM, respectively, as described (33, 34, 35). For cell treatment, the fatty acid stock was diluted to appropriate concentrations in 1% BSA containing complete growth medium and incubated at 37°C for 30 min for fatty acid conjugation to BSA. One percent of BSA-containing media was prepared by dissolving fatty acid–free BSA in complete growth medium at room temperature and further incubating at 37°C for 25 min. Pathophysiological and physiologically achievable concentration range of 200–400 μM PA were used in the present study and vehicle (Veh) treatment were <1% isopropanol in 1% BSA-containing medium. For PO, a standard concentration of 200 μM was used throughout the study.

### Characterization of cell death

Cell death was analyzed via assessing percent apoptotic nuclei, which represent structural markers of cell death. Percent cell death was quantified by characteristic nuclear morphology and visualized with the treatment of DNA binding fluorescent dye, 4′, 6-diamidine-2-phenylindole dihydrochloride (DAPI) (33). Briefly, cells were stained with DAPI (5 μg/ml) for 20–25 min at 37°C. Dying cells were characterized by membrane permeabilization and condensation and fragmention of nuclei were counted and presented as percent of total nuclei. Experiments were performed in triplicates and at least 200 cells were counted per well (33).

### Cell lysate preparation and immunoblot analysis

Cells were washed with ice cold PBS (1X) once and scraped from the plate using cell lysis buffer (50 mM Tris pH 7.4, 150 mM NaCl, 1 mM EDTA, 1 mM DTT, 1 mM Na_3_Vo_4_, 1 mM PMSF, 100 mM NaF, and 1% Triton x-100) supplemented with Halt protease and phosphatase inhibitor cocktail (# 78440, Thermo Fisher Scientific, MA). Collected cell lysates were incubated on ice for 30 min with interval vortexing to facilitate cell lysis, then centrifuged at 21,130 *g* for 20 min at 4°C, and the supernatant containing cellular protein was collected. Total protein quantification was performed using modified Lowry's method with Pierce 660 nm protein assay reagent (# 22660, Thermo Fisher Scientific, MA). A total of 10–20 μg protein were resolved on 10% or 12% polyacrylamide gel containing SDS and further transferred onto nitrocellulose membrane (Bio-Rad, CA) using a Bio-Rad wet transfer system. Nonspecific protein blocking was performed using either 5% skim milk or BSA in TBS-containing 0.1% tween 20 and incubated with primary antibody (1:1000) solution at 4°C overnight. The membranes were washed three times with TBS-containing 0.1% tween 20 and incubated with HRP-conjugated secondary antibody (1:5000 dilution) solution for 2 h at room temperature. Protein bands visualized using chemiluminescent ECL substrate (# 170-5061, Bio-Rad; # NEL104001, PerkinElmer, MA; or # A38554), Thermo Fisher Scientific, MA using Bio-Rad Chemidoc imaging system.

### Isolation of nuclear proteins

Cells were washed with PBS and scraped using buffer A (10 mM Hepes, 10 mM KCl, 0.1 mM EDTA, 0.1 mM DTT, 0.5% nonidet-P40 substitute (MilliporeSigma)) supplemented with protease inhibitor (Roche) and incubated on ice for 10 min. Cell lysate was centrifuged at 15,000 *g* for 3 min and supernatant was separated (which contains cytosolic protein). To the pelleted nuclear content, buffer B (20 mM Hepes, 0.4 M NaCl, 1 mM EDTA, 0.05 mM DTT, and 10% glycerol) containing protease inhibitor was added and incubated on ice with intermittent vortexing for 40 min. The nuclear content was centrifuged at 15,000 *g* for 5 min, and supernatant containing nuclear protein was collected and stored at −80°C until used.

### Quantitative real-time PCR analysis

Cells were lysed in the well using TRIzol reagent (# 15596018, Invitrogen, MA) and RNA was isolated according to manufacturer’s instruction. Isolated total RNA was quantified and checked for purity using Biotek Synergy plate reader. One microgram of total RNA was reverse transcribed into cDNA using random hexamers, RNase OUT (Invitrogen, MA), dNTPs, and Murine-MuLV reverse transcriptase (NEB, MA). Quantitative real-time PCR was performed using Light Cycler 480 SYBR Green I Master mix (# 04707516001, Roche, Basel, Switzerland) according to manufacturer instructions in a Bio-Rad Cfx Connect real-time system. Target genes and their primers are listed in Table 1.

**Table 1:** List of primers used

Target (mouse)	Forward (5′-3′)	Reverse (5′-3′)
IL-1β	GTCACAAGAAACCATGGCACAT	GCCCATCAGAGGCAAGGA
IL-6	CTGCAAGAGACTTCCATCCAGTT	AGGGAAGGCCGTGGTTGT
TNF-α	GGCTGCCCCGACTACGT	ACTTTCTCCTGGTATGAGATAGCAAAT
HPRT	CGTCGTGATTAGCGATGATG	ACAGAGGGCCACAATGTGAT
36B4	GGATCTGCTGCATCTGCTTG	GGCGACCTGGAAGTCCAACT

Target (human)	Forward (5′-3′)	Reverse (5′-3′)

IL-1β	GCCCTAAACAGATGAAGTGCTC	GAACCAGCATCTTCCTCA
IL-6	CTTCTCCACAAGCGCCTTC	CAGGCAACACCAGGAGCA
TNF-α	GGCAGTCAGATCATCTTCTCG	GGTTTGCTACAACATGGGCTA
18S	TCAACTTTCGATGGTAGTCGCCGT	TCCTTGGATGTGGTAGCCGTTTCT

IL, interleukin.

### Measurement of IL-1β

IL-1β release was detected using ELISA kit according to manufacturer’s instructions (eBioscience# 88701388; Invitrogen# 88726188). Briefly, cells were treated accordingly, and culture supernatant was collected and centrifuged to pellet down any floating cell or cellular debris. Uncoated ELISA plates were coated with capture antibody overnight at 4°C, washed three times, and blocked for 1 h at room temperature. One hundred microliters of cell culture supernatant prepared above was added to individual wells and incubated at room temperature for 2 h. Plates were then washed appropriately, antibody detection solution was added, and were further incubated at room temperature for 1 h. Plates were again washed, streptavidin conjugated–HRP was added and incubated for 30 min at room temperature. Plates were washed, and 3,3′,5,5′-tetramethylebenzidine solution was added and incubated at room temperature for 15 min. Finally, stop solution was added and absorbance was measured at 450 nm. Concentrations were calculated by generating an IL-1β standard curve.

### Statistical analysis

The data were analyzed using two-way ANOVA with Bonferroni posthoc test for comparison between multiple groups and Student’s *t* test for comparison between two groups using GraphPad Prism 9. The data were plotted as means and SEM using GraphPad Prism.

## Results

### PO protects against LPS-induced macrophage inflammation

**Fig 1:**
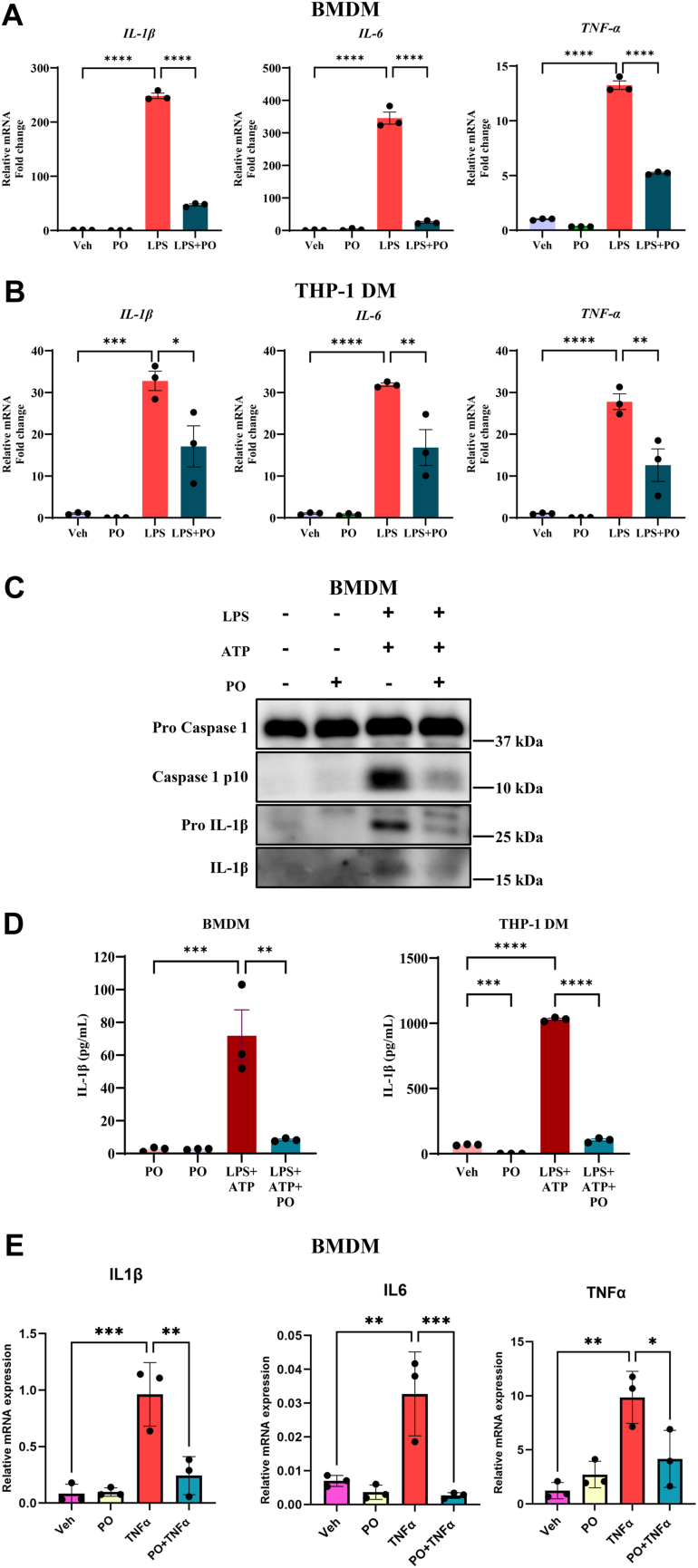
Palmitoleate prevents LPS-induced inflammation and inflammasome activation in macrophages. Mouse bone marrow–derived macrophages (BMDMs) and THP-1–derived macrophages (THP-1 DMs) were exposed with LPS (10 ng/ml) for 3 h to mimic inflammation, in vitro. LPS increased the expression of proinflammatory cytokines like *IL-1β*, *IL-6*, *and TNF-α* mRNA relative to control *HPRT* mRNA expression. Pretreatment of palmitoleate (PO), 200 μM, for 16 h significantly decreased the mRNA levels of proinflammatory cytokines in BMDMs, and THP-1 DM with LPS (A, B). BMDMs were exposed with LPS (10 ng/ml) for 3 h and ATP (5 mM) for 30 min showed increased protein expression of pro-IL-1β, IL-1β, and cleaved caspase 1 as measured by immunoblot. Pretreatment of PO for 16 h and then stimulation with LPS for 3 h followed by ATP for 30 min decreased the expression of pro-IL-1β, IL-1β, and active caspase 1 suggesting that PO prevents inflammasome activation (C). Increased IL-1β release into the culture supernatant with the exposure of LPS + ATP suggests inflammasome activation and treatment of PO significantly prevented the release of IL-1β into the culture supernatant, suggesting that palmitoleate has anti-inflammatory and anti-inflammasome properties in macrophages (D). BMDMs were exposed with TNFα (10 ng/ml) for 30 min to mimic inflammation, in vitro. TNFα increased the expression of proinflammatory cytokines like IL-1β, IL-6, *and*TNF-α mRNA relative to control HPRT mRNA expression. Pretreatment of palmitoleate (PO), 200 μM, for 16 h followed by TNFα for 30 min significantly decreased the mRNA levels of proinflammatory cytokines in BMDMs (E).The data represent the mean ± SEM for n = 3. ∗*P* < 0.05, ∗∗*P* < 0.01, ∗∗∗*P* < 0.001, and ∗∗∗∗*P* < 0.0001. IL, interleukin; LPS, lipopolysaccharide.

To determine the protective role of PO against inflammation, we used mouse BMDMs and stimulated them with LPS (10 ng/ml) for 3 h to induce inflammation. We observed that treatment of LPS to BMDMs increased mRNA expression of proinflammatory cytokines such as IL-1β, IL-6, and TNF-α (Fig. 1A). When BMDMs were pretreated with PO followed by LPS induction, we observed a significantly reduced expression of proinflammatory cytokines compared to LPS treated BMDMs (Fig. 1A). Similar to BMDMs, mouse macrophage cell RAW264.7 also responded to LPS (10 ng/ml) induction by increasing the mRNA synthesis of proinflammatory cytokines, and pretreatment of PO followed by LPS induction resulted in significant inhibition of proinflammatory cytokine mRNA synthesis (supplemental Fig. S1). To further validate the anti-inflammatory potential of PO, we differentiated human THP-1 monocytes to THP-1 DM in vitro using PMA. Similar to BMDMs and mouse macrophages, treatment of LPS (10 ng/ml) to THP-1 DMs upregulated the expression of proinflammatory cytokines IL-1β, IL-6, and TNF-α mRNAs compared to vehicle treated cells (Fig. 1B). Pretreatment of PO for 16 h followed by LPS stimulation showed significantly decreased mRNA expression of proinflammatory cytokines such as IL-1β, IL-6, and TNF-α compared to LPS alone treated THP-1 DMs (Fig. 1B). Together, these data suggest a potent anti-inflammatory role of PO against LPS-induced macrophage inflammation.

### PO prevents inflammasome-mediated activity in macrophages

We next tested the protective role of PO against LPS and ATP-induced NLRP3 inflammasome activation in the BMDMs. Using immunoblot analysis, we observed that LPS and ATP exposure resulted in dramatic increase in pro-IL-1β, mature IL-β, and cleaved caspase 1 protein levels compared to vehicle or PO-treated cells alone. Pretreatment of PO followed by LPS and ATP markedly decreased the levels of pro-IL-1β, active IL-1β and cleaved caspase 1p10 subunit, which could decrease the levels of mature IL-1β (Fig. 1C). We did not observe any changes in the levels of procaspase 1 with different treatment conditions in BMDMs. Similarly, BMDMs exposed to LPS and ATP showed a significant increase in the release of IL-1β protein levels into the culture supernatant as compared to vehicle treated BMDMs. Pretreatment of PO for 16 h followed by LPS and ATP exposure significantly decreased the protein levels of IL-1β in the culture supernatant (Fig. 1D).

NLRP3 inflammasome priming with LPS and activation with ATP for 30 min to THP-1 DM also showed significantly increased release of IL-1β protein into the cell culture supernatant compared to vehicle or PO alone treated cells (Fig. 1D). Pretreatment of PO for 16 h before LPS priming and activation with ATP (30 min before sample collection) showed a significant decrease in IL-1β protein levels in the culture supernatant of THP-1 DMs suggesting an inhibition of NLRP3 inflammasome activation (Fig. 1D). Further, we also tested the protective role of PO against TNFα-induced inflammation in BMDMs. We observed that treatment of TNFα (10 ng/ml) to BMDMs for 30 min resulted in an increased mRNA expression of proinflammatory cytokines such as IL-1β, IL-6, and TNF-α (Fig. 1E). Pretreatment of PO followed by TNFα exposure significantly decreased the mRNA levels of IL-1β, IL-6, and TNFα (Fig. 1E) Together, these data suggest that supplementation of PO prevents the activation of LPS or TNFα-induced inflammatory responses in BMDMs and PO also prevents LPS/ATP-induced NLRP3 inflammasome activation in both mouse and human macrophages.

### PO inhibits inflammation in trophoblasts

**Fig 2:**
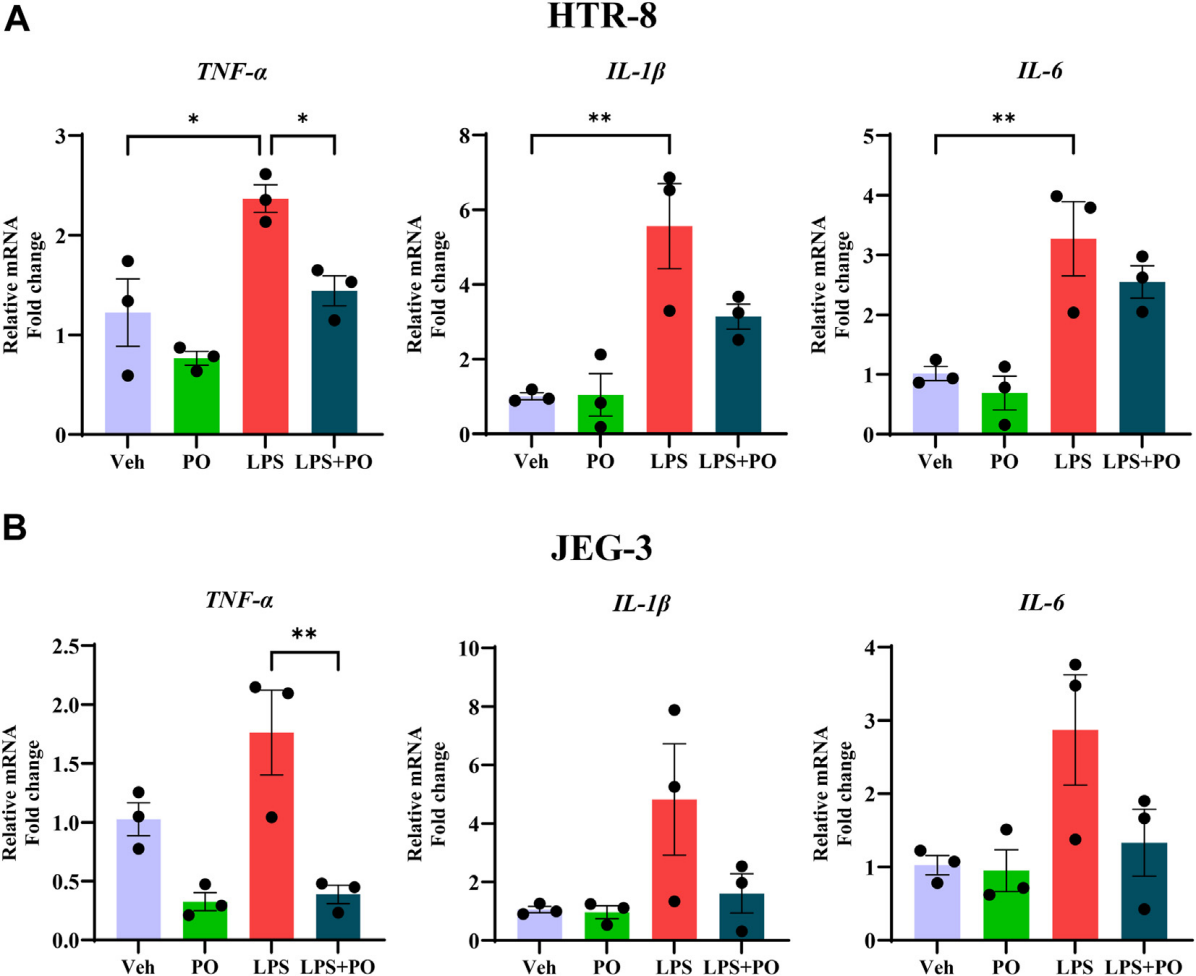
Palmitoleate (PO) prevents LPS-induced inflammation in trophoblasts. HTR-8 cells (A) or JEG-3 cells (B) with 1 μg/ml of LPS for 4 h to mimic maternal systemic inflammation in vitro. LPS increased the expression of proinflammatory cytokines *IL-1β*, *IL-6*, *and TNF-α* mRNA relative to control *18S rRNA* expression in trophoblasts. Pretreatment of 200 μM PO for 16 h followed by LPS for 4 h prevents LPS alone induced inflammation as evidenced by decreased the mRNA levels of proinflammatory cytokine TNF-α in HTR-8 (A) and JEG-3 cells (B). The data represent the mean ± SEM for n = 3. ∗*P* < 0.05, ∗∗*P* < 0.01. IL, interleukin; LPS, lipopolysaccharide.

Next, we validated the protective role of PO in trophoblasts of placental origin, affected during MO (36). Treatment of LPS (1 μg/ml) for 4 h to first trimester–derived trophoblasts, HTR-8 cells, showed a significantly increased mRNA levels of IL-1β, IL-6, and TNF-α (Fig. 2A). However, pretreatment of PO for 16 h followed by LPS exposure only prevented the mRNA expression of TNF-α (Fig. 2A). We also observed, LPS exposure (1 μg/ml) for 4 h to JEG-3 cells, another trophoblast showed an increase in the mRNA expression of TNF-α and pretreatment of PO followed by LPS exposure decreased the expression of TNF-α mRNA expression relative to 18S compared to LPS alone in JEG-3 cells (Fig. 2B). However, LPS exposure to JEG-3 cells showed a trend in increase toward the expression of IL-1β and IL-6 mRNAs (Fig. 2B). Together, supplementation of PO partially prevents placental trophoblast-derived cells from LPS-induced inflammatory processes.

### PO decreases the expression of Pro-IL-1β in trophoblasts

**Fig 3:**
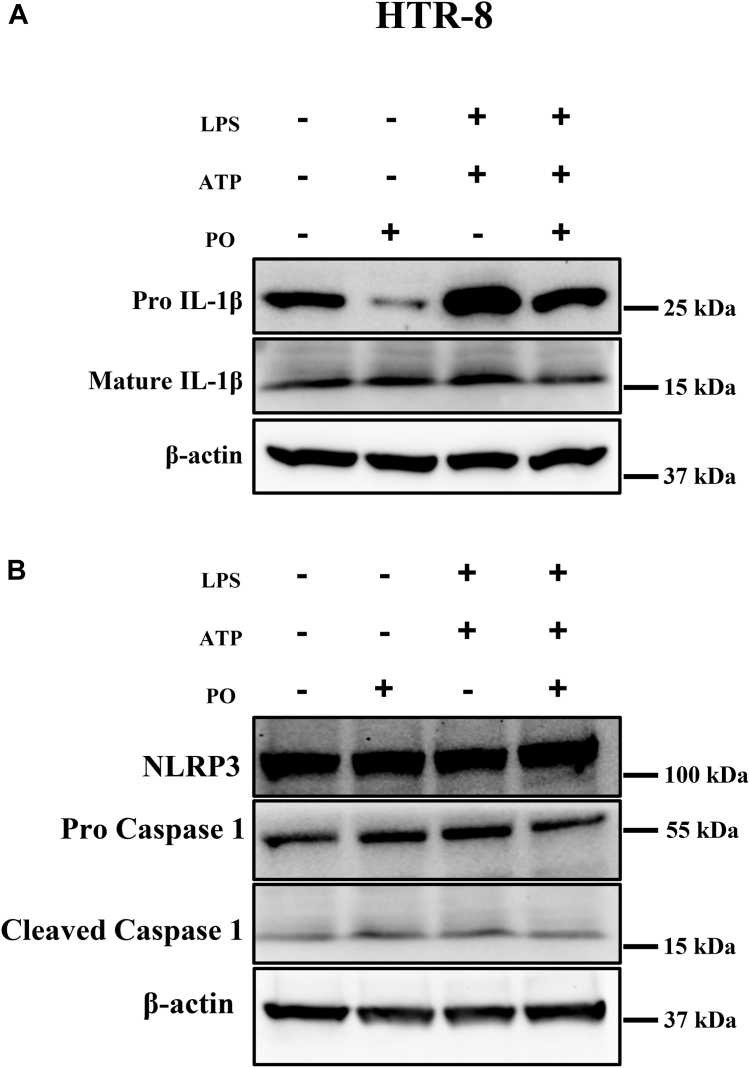
Palmitoleate decreases the expression of Pro-IL1β in trophoblasts. HTR-8 cells were exposed with LPS (1 μg/ml) for 4 h and ATP (5 mM) for 30 min showed increased protein expression of pro-IL-1β, and mature IL-1β measured by immunoblot. Pretreatment of PO for 16 h and then sequential treatment with LPS (4 h) + ATP (30 min), decreased the expression of Pro-IL-1β, and mature IL-1β. Actin levels were unchanged in all the treatment conditions (A). Levels of NLRP3, procaspase 1 and cleaved and active caspase 1 were also unchanged with the treatment of LPS + ATP, LPS + ATP + PO compared vehicle-treated cells (B). The images are representative of 3–4 independent experiments. IL, interleukin; LPS, lipopolysaccharide; NLRP3, NACHT, LRR, and PYD domain–containing protein 3.

HTR-8 cells were treated with LPS 1 μg/ml for 4 h and ATP for 30 min to activate NLRP3 inflammasome. Treatment of LPS + ATP increased the expression of pro-IL-1β and a subtle increase in mature IL-1β compared to vehicle-treated cells. Pretreatment of PO for 16 h by itself decreased the protein levels of pro-IL-1β. Further, pretreatment of PO followed by LPS + ATP exposure substantially blocked the protein levels of pro-IL-1β compared to LPS + ATP alone treatment (Fig. 3A). LPS + ATP-induced increase in mature IL-1β was also decreased with the treatment of PO. There was no change in the levels of control protein, β-actin among the different treatment conditions tested. Next, we tested for the levels of NLRP3 protein and inflammasome activation downstream target, cleaved caspase 1 with LPS + ATP and with PO pretreatment followed by LPS + ATP stimulation. We did not observe any changes in the NLRP3, procaspase 1, cleaved caspase 1, and β-actin compared to vehicle-treated trophoblasts (Fig. 3B). These data suggest that supplementation of PO decreases the expression of pro-IL-1β in trophoblasts.

### PO alters LPS-induced activation of NF-κB pathway

**Fig 4:**
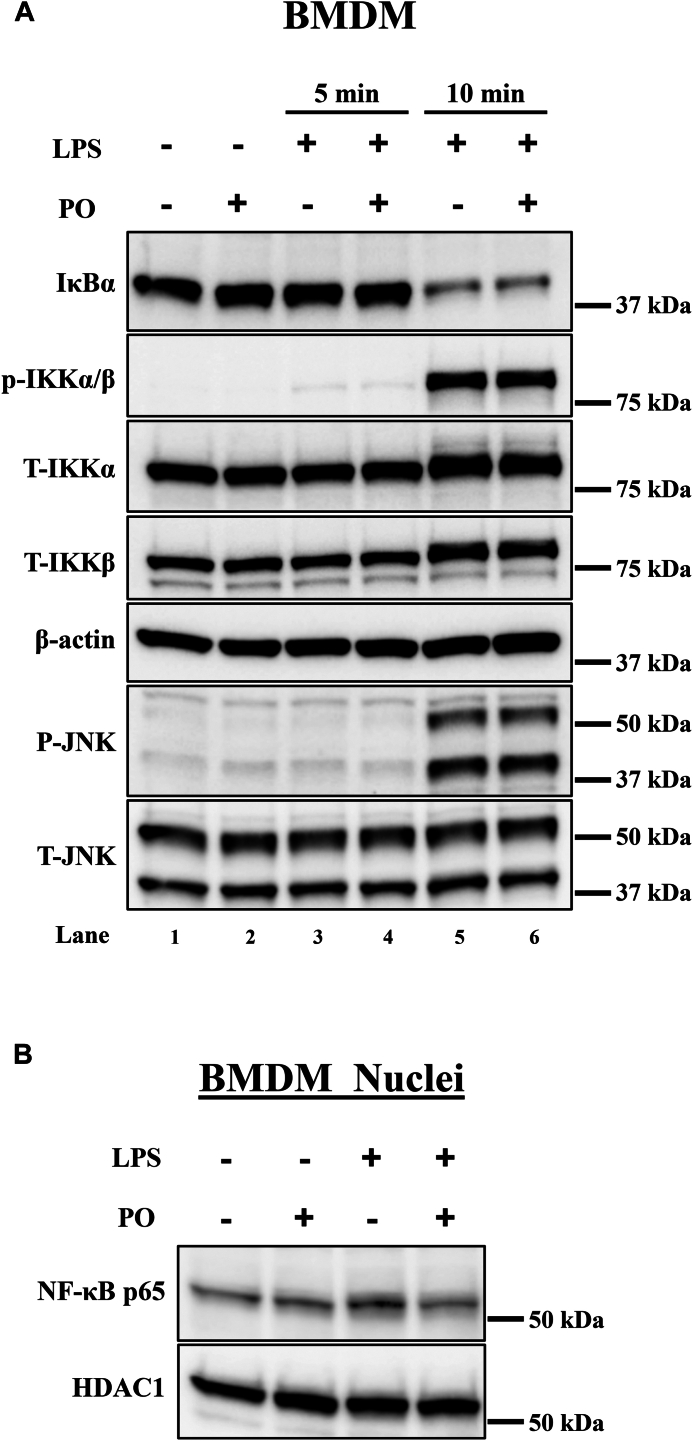
Palmitoleate (PO) protects against LPS-induced inflammation by blocking NF-κB nuclear translocation or activation. BMDMs were stimulated with LPS (10 ng/ml) alone or in presence of PO (pretreatment for 16 h at 200 μM) for 5–10 min and analyzed for NF-κB signaling pathway activation. Immunoblot analysis showed decreased levels of IκBα after 10 min of LPS exposure (A). Pretreatment of PO however did not block the degradation of IκBα (A). LPS exposure (5–10 min) also increased phosphorylated forms of IKKα/β and PO did not alter the levels of phospho-IKKα/β after 5–10 min. Immunoblot analysis also showed that total IKK-α, IKK-β, and β-actin levels remained unchanged in all the treatment conditions tested (A). We also observed increased phosphorylation of JNK following LPS exposure, and treatment of palmitoleate did not change the levels of phosphorylated JNK (A). Total JNK level remained the same in all experimental conditions (A). NF-κB p65 nuclear translocation were tested by immunoblot following LPS exposure in BMDMs and observed to be increased in the nuclei, suggesting increased nuclear translocation of p65 subunit. Pretreatment of PO for 16 h followed by LPS exposure showed decreased nuclear levels of p65 subunit in BMDMs (B). Histone deacetylase 1 was used as nuclear loading control and showed no change under any treatment conditions (B). BMDM, bone marrow–derived macrophage; JNK, c-Jun N-terminal kinase; LPS, lipopolysaccharide.

We next sought to test whether PO may have role in blocking LPS-induced activation of NF-κB signaling pathway, thereby preventing LPS-related inflammatory response in BMDMs. Activation of canonical NF-κB signaling pathway involves phosphorylation of IKKα/β and subsequent degradation of IκBα via ubiquitin-mediated proteasomal degradation. BMDMs were exposed with 10 ng/ml of LPS for 5 to 10 min and we observed a simultaneous degradation of IκBα and drastic increase in the levels of phosphorylated IKKα/β after 10 minutes of exposure (Fig. 4A, lane 5). However, we did not observe any changes with the pretreatment of 200 μM of PO and LPS in both IκBα and phospho-IKKα/β levels compared to LPS alone treated macrophages (Fig. 4A, lane 6). Further, the levels of total forms of IKKα and IKKβ did not change after 5 or 10 min of exposure with LPS or LPS + PO-treated cells (Fig. 4A). We also observed that after 10 min of LPS exposure, a substantial increase in the levels of phosphorylated JNK (Fig. 4A, lane 5) and treatment of PO followed by LPS exposure did not alter the levels of phospho-JNK compared to LPS alone treated cells (Fig. 4A, lane 6). There were no changes in the levels of total-JNK and β-actin in BMDMs with different treatment conditions (Fig. 4A). Degradation of IκBα results in increased NF-κB p50 and p65 subunits translocation into the nucleus and transcriptional activation of NF-κB downstream targets such as proinflammatory cytokines. Therefore, we next tested whether PO modulates the nuclear translocation of NF-κB p65 subunit via immunoblot and observed that nuclear levels of p65 proteins were increased with LPS exposure in BMDM cells after 15 min (Fig. 4B). Interestingly, treatment of PO followed by LPS to BMDMs decreased the nuclear levels of NF-κB p65 subunit compared to LPS alone treated cells (Fig. 4B), and the nuclear levels of histone deacetylase 1 were unchanged in vehicle, PO, LPS alone, or LPS + PO treatment conditions (Fig. 4B). These data suggest that PO blocks the nuclear translocation of NF-κB p65 subunit during LPS-induced inflammatory response in BMDMs.

### LPS and PA-induced cell death in BMDMs

**Fig 5:**
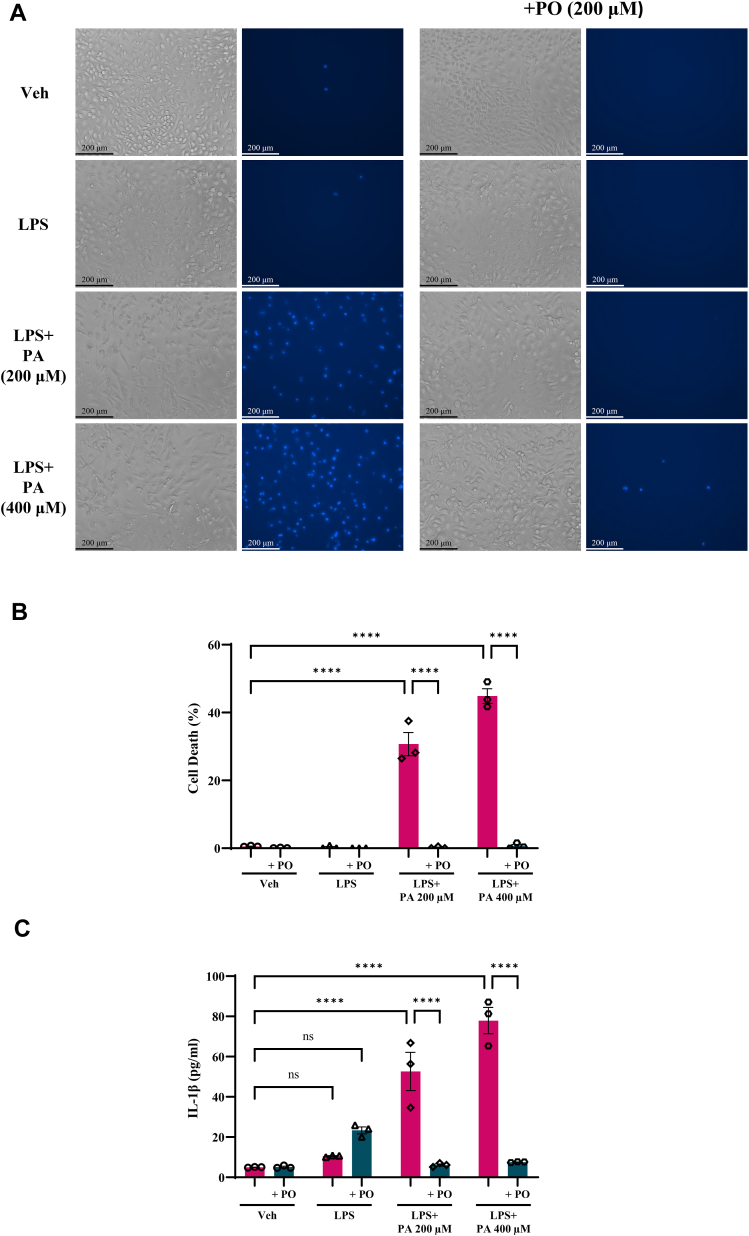
Palmitoleate protects against LPS and palmitate mediated cell death and IL-1β release in BMDMs. We exposed BMDMs with LPS for 3 h and then treated with palmitate (PA, 200–400 μM) for 16 h and percent cell death were measured. LPS alone did not increase percent cell death, and treatment of PA post LPS exposure resulted in a significant increase in percent cell death compared to vehicle (Veh) treated BMDMs (A, B). Pretreatment of palmitoleate (PO, 200 μM) followed by LPS and PA treatment, significantly prevented LPS + PA-induced increase in percent cell death in BMDMs (A, B). Release of IL-1β in cell culture supernatant showed that LPS and palmitate exposure to BMDMs results in a significant increase in mature IL-1β release to culture supernatant compared to Veh cells. Pretreatment of PO prevented IL-1β release in response to LPS and palmitate coexposure (C). The image shown is representative images from n = 3. Data represents means ± SEM, ∗∗∗∗*P* < 0.0001. BMDM, bone marrow–derived macrophage; IL, interleukin; LPS, lipopolysaccharide.

Obesity is associated with increased circulating levels of saturated FFAs (36) and LPS (a consequence of increased gram-negative bacterial composition in the gut microbiota). So, we next asked whether PO can protect against LPS and FFA-induced cellular death and inflammation. BMDMs were pretreated with PO for 16 h and then exposed to LPS (10 ng/ml) for 3 h followed by 16 h of 200–400 μM PA with and without PO, and percent cell death was measured using nuclear morphological changes by DAPI staining. From our earlier work, we know that LPS alone does not induce cell death (33). However, treatment of 200–400 μM PA following LPS exposure resulted in increased percent cell death (33). Fig. 5A shows representative images of phase contrast and DAPI stained nuclear morphological changes. We observed a marked increase in nuclear DNA condensation and fragmentation with the treatment of LPS + PA 200 or 400 μM compared to LPS or vehicle-treated BMDMs (Fig. 5A). We also observed that pre/cotreatment of PO diminished the number of DAPI-positive cells compared to LPS + PA-treated BMDMs (Fig. 5A). We further quantitated percent cell death by counting the number of cells that showed nuclear morphological changes with respect to the total number of cells using epifluorescence microscopy. LPS + PA 200 μM and LPS + PA 400 μM showed a significant increase in percent cell death and pretreatment/cotreatment of 200 μM PO significantly prevented LPS + PA-induced cell death (Fig. 5B). These data suggest that PO has a unique protective role against LPS + PA-induced cell death in BMDMs. To further elucidate whether the mechanism of LPS + PA-induced cell death involves inflammasome activation, we measured the levels of IL-1β in the BMDM culture supernatant and found that LPS + PA significantly increased the release of mature IL-1β and pretreatment/cotreatment of PO with LPS + PA to BMDMs showed significantly reduced release of IL-1β into the culture supernatant (Fig. 5C). Supplementation of PO significantly decreases the levels of IL-1β release into the culture media in both 200 and 400 μM of PA + LPS-treated BMDMs. These data further suggest that PO prevents LPS + PA-induced inflammasome activation and release of mature IL-1β in BMDMs.

### PO prevents LPS + PA-induced NLRP3 inflammasome activity in BMDMs

**Fig 6:**
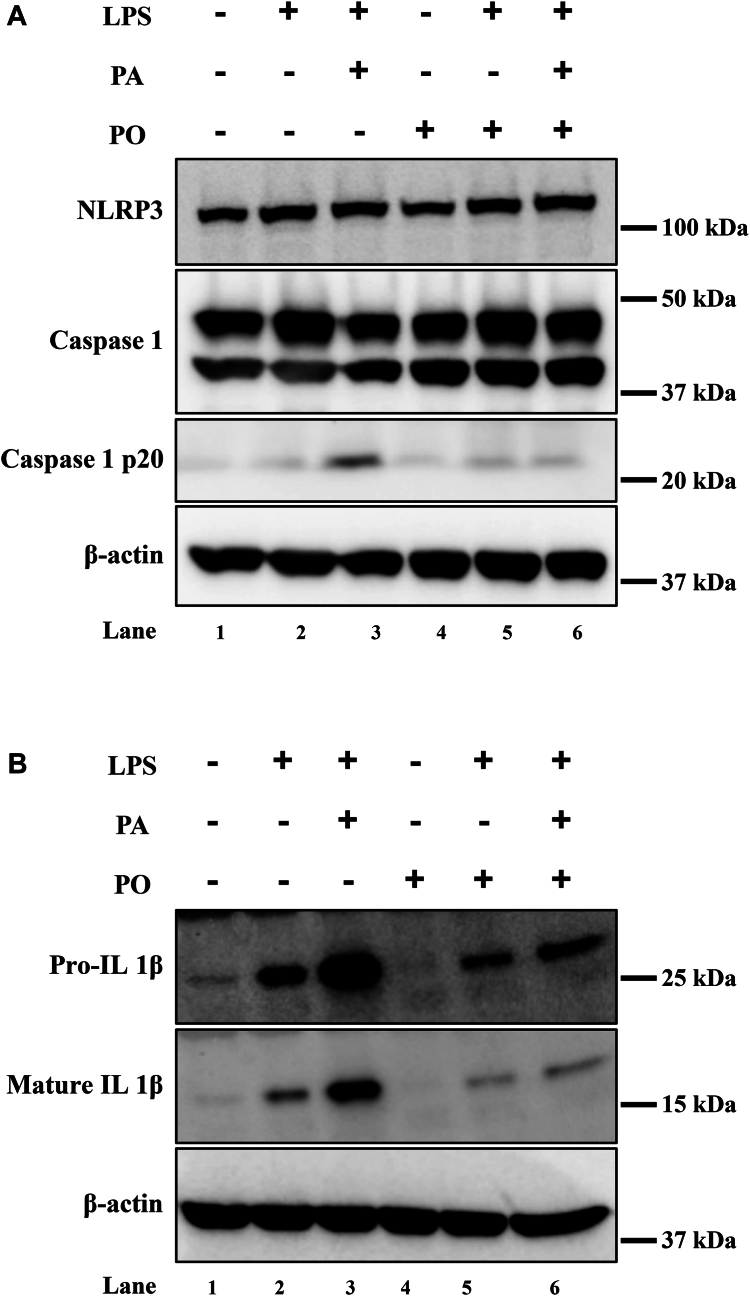
Palmitoleate inhibits inflammasome-mediated caspase 1 and pro-IL-1β cleavage. Immunoblot analysis of BMDMs exposed with LPS and palmitate (PA) resulted in the cleavage of caspase 1 to cleaved caspase 1 p20 subunit (A, lane 3) and pretreatment of PO blocked cleavage of caspase 1 (A, lane 6). BMDM exposed to LPS and PA, showed an increase in both the pro and mature form of IL-1β compared to vehicle-treated cells (B, lane 1 and 3). Pretreatment of PO followed by LPS and PA resulted in a decreased levels of both pro and mature IL-1β protein compared to LPS and palmitate-treated cells (B, lane 3 and 6). NLRP3 and β-actin levels remains unchanged with different treatment conditions tested. BMDM, bone marrow–derived macrophage; IL, interleukin; LPS, lipopolysaccharide.

We next measured the downstream mediators of NLRP3 inflammasome activation in BMDMs. Immunoblot analysis of NLRP3 and caspase-1 protein levels did not show any changes with the treatment of PO alone, LPS alone, LPS + PA (400 μM), and LPS + PA + PO compared to control BMDMs (Fig. 6A). However, we observed increased active caspase 1 p20 subunit in BMDMs exposed to LPS + PA compared to control and LPS or PO alone treated BMDMs (Fig. 6A, lane 3). There was a substantial reduction in the levels of active caspase 1 p20 with the pretreatment/cotreatment of PO and LPS + PA exposure (Fig. 6A, lane 6). BMDMs exposed to LPS increased the levels of pro-IL-1β and mature IL-1β and both of their levels were further enhanced with the addition of 400 μM PA (Fig. 6B, lane 2 and 3, respectively). However, treatment of PO decreased the levels of pro-IL-1β and mature IL-1β levels in BMDMs (Fig. 6B, lane 5 and 6). Actin was used to measure control loading and its levels did not change with the different treatment conditions. These data suggest that PO prevents the molecular events downstream of NLRP3 inflammasome activation.

### PO protects against MAPK activation in BMDMs

**Fig 7:**
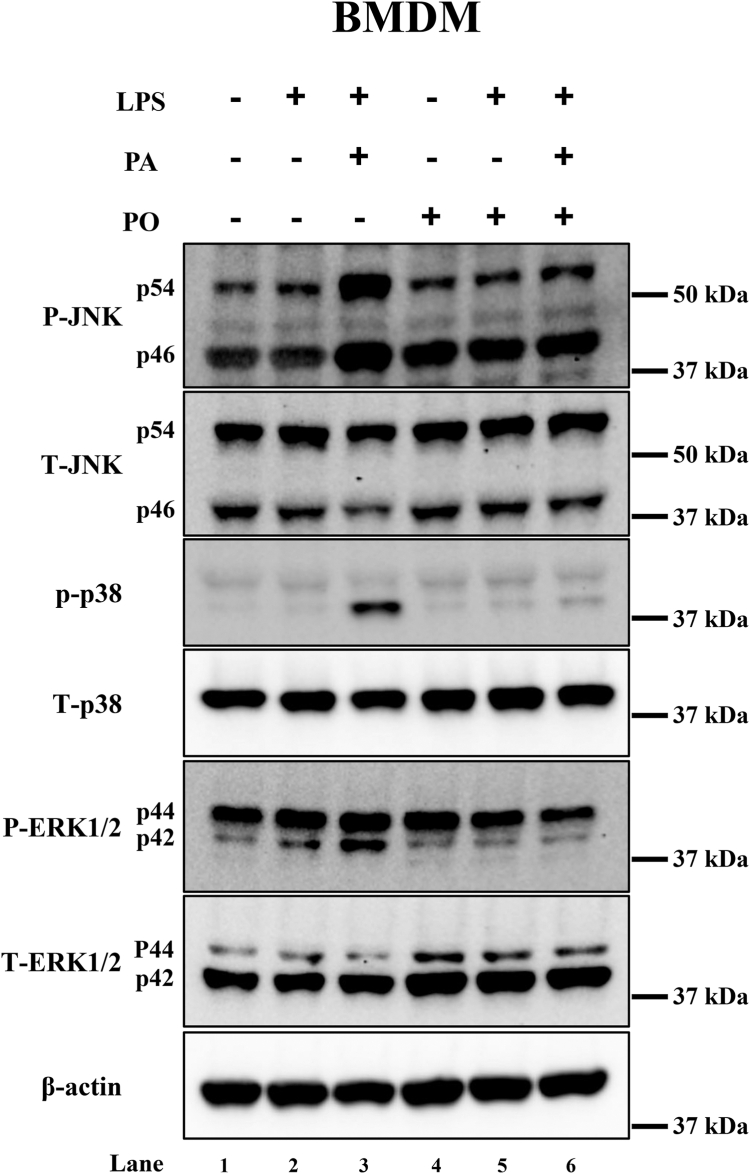
LPS and palmitate (PA) activates MAPKs in BMDMs. Immunoblot analysis of BMDMs exposed with LPS (10 ng/ml) for 3 h and then palmitate (PA, 400 μM) for 16 h showed MAPKs activation as evidenced by robust increase in the levels of phospho-JNK, phospho-p38, and phospho-ERK1/2 (lane 3) compared LPS alone or vehicle-treated cells (lane 1 and 2). Pretreatment of palmitoleate (200 μM, PO) followed by LPS and PA sequential exposure blocked phosphorylation status of all three MAPKs (p-JNK, p-p38, and p-ERK levels) compared to LPS and palmitate treated BMDMs (lane 6). Total JNK, p38, ERK1/2, and β-actin levels remained similar in all experimental conditions tested. BMDM, bone marrow–derived macrophage; IL, interleukin; JNK, c-Jun N-terminal kinase; LPS, lipopolysaccharide; MAPK, mitogen-activated protein kinase.

To further investigate the signaling mechanism of PO protection against inflammasome activation, we sought to test the role and activation of MAPKs, like JNK, ERK, and p38 MAPKs, which are known to be involved in the activation of inflammasome. BMDMs showed increased phosphorylation of JNK in both p56 and p46 kDa subunits with the exposure of LPS + PA compared to control BMDMs and pretreatment/cotreatment of PO to LPS + PA-exposed BMDMs showed a decreased levels of both phospho-JNK subunits (Fig. 7, lane 3 and 6). There were no changes in the total forms of JNK p56 and p46 subunits. Similar to JNK, we observed an increase in the levels of phosphorylated form of p38 MAPK (p-p38) in LPS + PA-treated BMDMs compared to controls, suggesting the activation of p38 MAPK and pretreatment/cotreatment of PO with LPS + PA-blocked the activation of p38 MAPK (Fig. 7, lane 3 and 6, respectively). Total p38 levels were also unchanged in different treatment conditions in BMDMs. We further observed the activation of ERK1/2 by way of measuring phosphorylated levels of ERK1/2, a stress activatable kinase and found that LPS increased phosphorylation of p42 subunit of ERK, which was further increased in LPS + PA-treated BMDMs compared to control BMDMs (Fig. 7, lane 2 and 3, respectively). Pretreatment/cotreatment of PO decreased the phosphorylated levels of ERK p42 subunit in BMDMs (Fig. 7, lane 5 and 6) compared to LPS or LPS + PA-treated BMDMs. Total ERK p42 and p44 subunits levels were unchanged, and actin was used to show control loading and were unchanged in different treatment conditions of BMDMs. These data suggest that LPS + PA-induced activation of MAPKs was prevented with the supplementation of PO.

## Discussion

**Fig 8:**
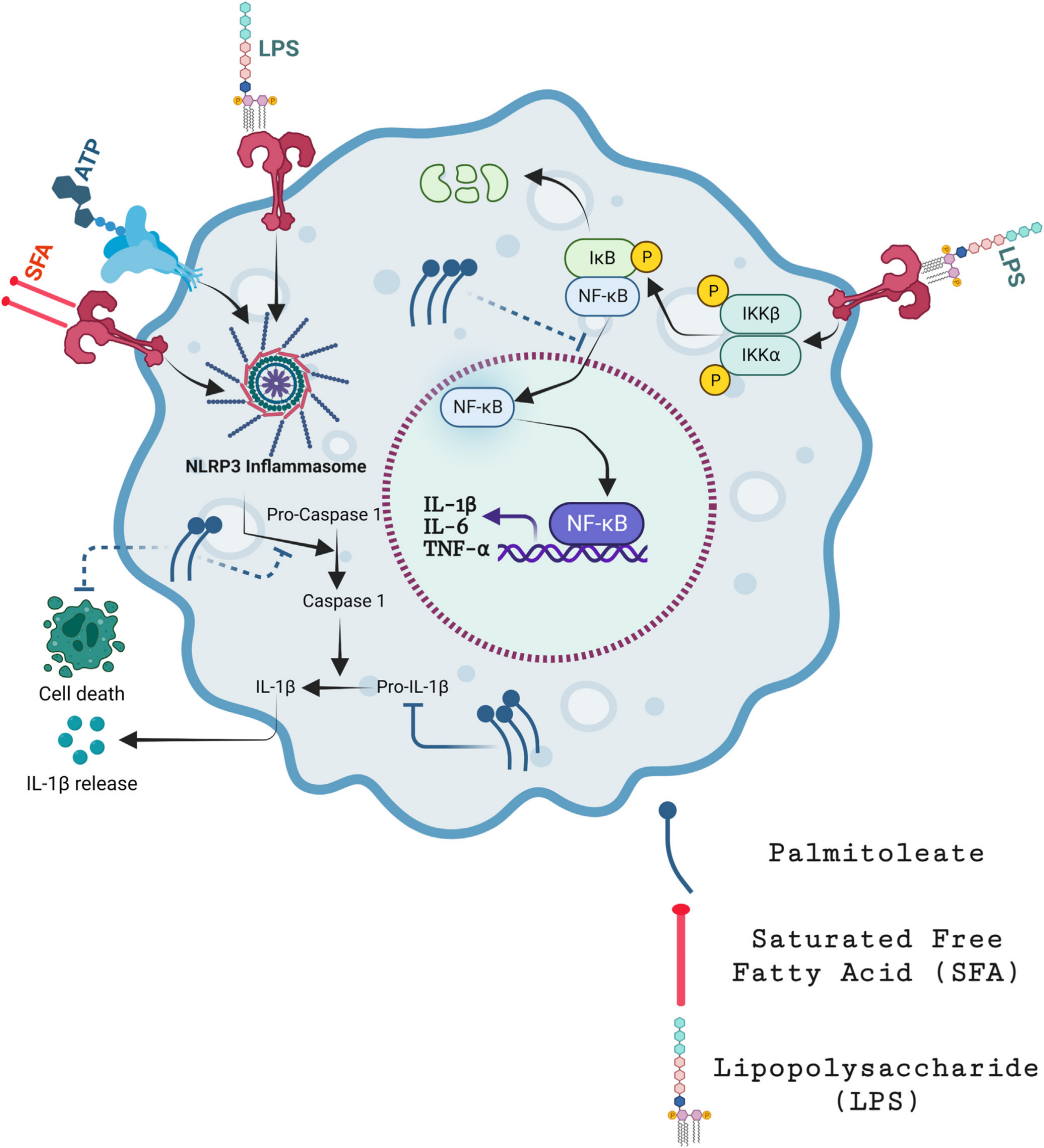
Schematic diagram on the protective role of palmitoleate against inflammation and inflammasome activity. Palmitoleate blocks NF-κB p65 subunit nuclear translocation, thereby blocking LPS-mediated activation of NF-κB pathway and proinflammatory cytokine gene upregulation. Palmitoleate also blocks NLRP3 inflammasome-mediated cleavage of procaspase 1 and pro-IL-1β to active caspase 1 p10/p20 subunits and IL-1β, respectively, thereby preventing LPS and saturated free fatty acid–induced cellular death and inflammation. Further, palmitoleate also prevents LPS + palmitate (PA)-induced cellular MAPK and inflammatory responses. Illustration was made using BioRender. IL, interleukin; LPS, lipopolysaccharide; MAPK, mitogen-activated protein kinase; NLRP3, NACHT, LRR, and PYD domain–containing protein 3.

The present study demonstrated that PO, an omega-7 monounsaturated fatty acid as a protective nutrient compound which prevents inflammation, inflammatory responses, inflammasome-mediated downstream events such as cell death. The principal findings of this study show that supplementation and cotreatment of PO protects against: 1) LPS and LPS + ATP-induced inflammatory response and inflammasome activity in mouse BMDMs; 2) LPS-induced inflammation in human THP-1 DMs and human trophoblasts; 3) LPS + PA-induced cell death and inflammasome activity in BMDMs; and 4) LPS and LPS + PA-induced inflammation dependent cell death potentially by preventing activation of (MAPK) in BMDMs (Fig. 8).These key signaling mechanisms of PO protection is described in a schematic diagram in Fig. 8.

LPS, a PAMP, which act on pathogen recognition receptor is well known to be increased in obesity and MO due to the intestinal barrier dysfunction and gut microbial dysbiosis (37, 38, 39). We used LPS in our in vitro culture models and showed that exposure of LPS induced proinflammatory cytokine expression and PO protected against LPS-induced inflammatory response in macrophages but not so much in HTR-8 and JEG-3, possibly due to the large of amount of LPS (1 ug/ml) used to induce inflammation in placental trophoblasts compared in macrophages (10 ng/ml), both BMDMs and THP-1 DM. Further, BMDMs express high levels of P2X_7_ receptor that mediate NLRP3 inflammasome-dependent secretion of IL-1β release with LPS and ATP exposure (40). PO exerts its protective effect against LPS + ATP- or LPS + PA-induced activation of inflammasome as observed with an increase in caspase 1 p20 subunit. LPS exposure alone does not increase the levels of cleaved caspase 1 levels in macrophages which reconfirms that both priming with PAMP (LPS exposure) and activation of NLRP3 inflammasome with ATP or PA are required for inflammasome activation (41). In response to both stimuli on the activation of inflammasome, PO dramatically prevented the increased levels of caspase 1 p10 and p20 subunits. These data suggest that PO is an anti-inflammatory monounsaturated fatty acid, which can be endogenously synthesized by the stearoyl CoA desaturase enzymes. However, it was observed that MO is associated with decreased levels of PO in the placenta and circulation. In contrast, placental stearoyl CoA desaturase1 expression were shown to be slightly increased in obese placenta compared to lean control (22).

We have earlier demonstrated that LPS aggravates PA-induced trophoblast lipoapoptosis and treatment of PO and oleate prevents PA-induced trophoblast lipoapoptosis (33). In the present study, we extended our findings to BMDMs and observed that LPS + PA induce cell death and that involves the activation of caspase 1. NLRP3 inflammasome can activate caspase 1 and can induce pyroptosis in cells. We observed the downstream markers for NLRP3 inflammasome activation and protection with the supplementation of PO. Also, PO blocks inflammation caused by LPS by decreasing the levels of TNF-α in the placental trophoblasts. However, further studies are required to test the critical role of inflammasome activation in PO protection against LPS + PA-induced cell death and in the aggravation of trophoblast lipoapoptosis.

Markers of inflammation and inflammasome activation were well observed in human or mouse-derived macrophages with the treatment of LPS, LPS + PA, and LPS + ATP. A key marker for inflammation and inflammasome activation was IL-1β release and increased cleaved caspase 1 were not observed in human trophoblasts. Future studies are needed to test this function in human trophoblasts isolated from term placenta. First trimester placental trophoblast cells, HTR-8 increased IL-1β, IL-6, and TNF-α mRNA expression with LPS and treatment of PO only prevented TNF-α expression, however JEG-3 cells which are third trimester choriocarcinoma–derived trophoblasts showed only a trend toward increase in the expression of proinflammatory cytokines like IL-1β and IL-6 could have difference in signaling response compared to first trimester–immortalized trophoblasts. Further, decreased TNF-α with the supplementation of PO in trophoblasts suggested the importance of NF-κB pathway, which could be independent mediator of blocking inflammasome activation observed in macrophages. LPS activates NF-kB and enhances nuclear translocation for the induction of proinflammatory cytokines. It is not clear how PO only prevents TNF-α but not the other proinflammatory cytokines in trophoblasts and that requires further investigation.

NF-κB signaling pathway is well known to activate proinflammatory cytokines and cell survival signals. We did not observe any changes in the upstream activators of NF-κB with and without PO supplementation in LPS exposed macrophages. However, we did observe changes in the nuclear activation of NF-κB p65 subunit with LPS, and supplementation of PO decreased the nuclear levels of p65 subunit. LPS signals via TLR4-dependent activation of NF-κB to induce its transcriptional targets within 10–30 min of exposure in BMDMs and peritoneal primary macrophages (42). We observed nuclear activation of p65 subunit 15 min post-LPS exposure and PO blocked the nuclear activation. However, further studies are required to test the expression of downstream transcriptional targets of NF-κB with the supplementation of PO. MAPK p38 is a known regulator of NF-κB and supplementation of PO prevents the activation of p38 (43, 44, 45, 46). In addition, PO also prevented the activation of JNK and ERK phosphorylation with LPS + PA treatment conditions. Blocking the activation of these MAPK could shed mechanistic role of NF-kB nuclear activation and needs further investigations.

Cotreatment of PA and LPS is known to induce ER stress which thereby induces the proinflammatory response in macrophages (47). We have earlier shown that PO protects against Zika virus infection-induced endoplasmic reticulum stress in the trophoblasts and in the present study PO protection against LPS + PA-induced JNK, ERK, and p38 MAPK activation could involve prevention of ER stress mediators (48, 49). For example, JNK can be activated via phosphorylation by IRE1α, which can be activated by ER stress. Further, JNK activation is also reported to be critical for the activation of NLRP3 inflammasome (50, 51, 52). Inhibition of JNK activation with PO supplementation by blocking ER stress could be a potential mechanism of protection. Nevertheless, genetic knock down studies, siRNA, or the use of small molecular inhibitors would provide further insights in the mechanism of PO protection which are currently underway.

In conclusion, treatment of PO prevents inflammation, inflammatory response caused due to LPS and LPS + ATP in mouse BMDMs, human THP-1–derived macrophages and trophoblasts. Interestingly, PO prevents the molecular activities downstream of inflammasome activation in mice and human-derived macrophages. PO also protects against LPS and FFA-induced cell death and MAPK activation. The mechanism of PO protection against inflammation and NLRP3 inflammasome activities might possibly be due to the road block on the activation of MAPK.

## Data availability

All the data generated or analyzed during this study are included in this article and in supplementary information files.

## Supplemental data

This article contains supplemental data.

## Conflict of interest

The authors declare that they have no conflicts of interest with the contents of this article.

## Supplemental data

Supplemantal Data
