# Supplementary material for: Palmitoleate protects against lipopolysaccharide-induced inflammation and inflammasome activity
Source: J Lipid Res. 2024 Oct 11;65(11):100672. doi: 10.1016/j.jlr.2024.100672 (PMC11585775; doi:10.1016/j.jlr.2024.100672)
Supplement: Supplemantal Data [file mmc1.docx]

**Palmitoleate protects against Lipopolysaccharide-induced Inflammation and Inflammasome Activity**

Prakash Kumar Sahoo^1^, Aiswariya Sekar^1^, Baolong Liu^1,2^, Jiujiu Yu^1,3^, Sathish Kumar Natarajan^1,4,5^*

^1^Department of Nutrition and Health Sciences, University of Nebraska-Lincoln, Lincoln, NE, USA; ^2^ Shaanxi Key Laboratory of Molecular Biology for Agriculture, College of Animal Science and Technology, Northwest A&F University, Yang ling, Shaanxi, China; ^3^Department of Nutrition, Case Western Reserve University, Cleveland, OH, USA; ^4^College of Allied Health Professions Medical Nutrition Education, University of Nebraska Medical Center, Omaha, NE, USA; ^5^Department of Biochemistry, University of Nebraska-Lincoln, Lincoln, NE, USA.

*Address for Correspondence: Sathish Kumar Natarajan, PhD

Assistant Professor, Department of Nutrition & Health Sciences

University of Nebraska-Lincoln

229 Filley Hall, Lincoln, NE 68583-0806

Tel: +1 402-805-7520

E-mail: [snatarajan2@unl.edu](mailto:snatarajan2@unl.edu)

ORCID: orcid.org/0000-0001-7491-8592

Running title: Palmitoleate prevents inflammation

Key words: Mono-unsaturated fatty acids, macrophages, trophoblasts, placenta, obesity, mitogen-activated protein kinase, pregnancy

The project described was supported by the United States Department of Agriculture (USDA)-National Institute of Food and Agriculture (NIFA) Standard Grant (2023-67017-40223); Revision Award from Agriculture Research Division, Institute of Agriculture Natural Resources; University of Nebraska-Lincoln, Nebraska Agricultural Experimental Station with funding from the Hatch Act (Accession #7005822) , USDA Multistate groups W5002, and W5122; University of Nebraska Collaborative Research Seed Grant Funding and Nebraska Center for Prevention of Obesity Diseases, the National Institute of General Medical Sciences Grant (P20GM104320) and U54 GM115458, which funds the Great Plains IDeA-CTR Network (all to SKN). The contents of this manuscript are solely the responsibility of the authors and does not necessarily represent the official views of the USDA and National Institutes of Health.

**
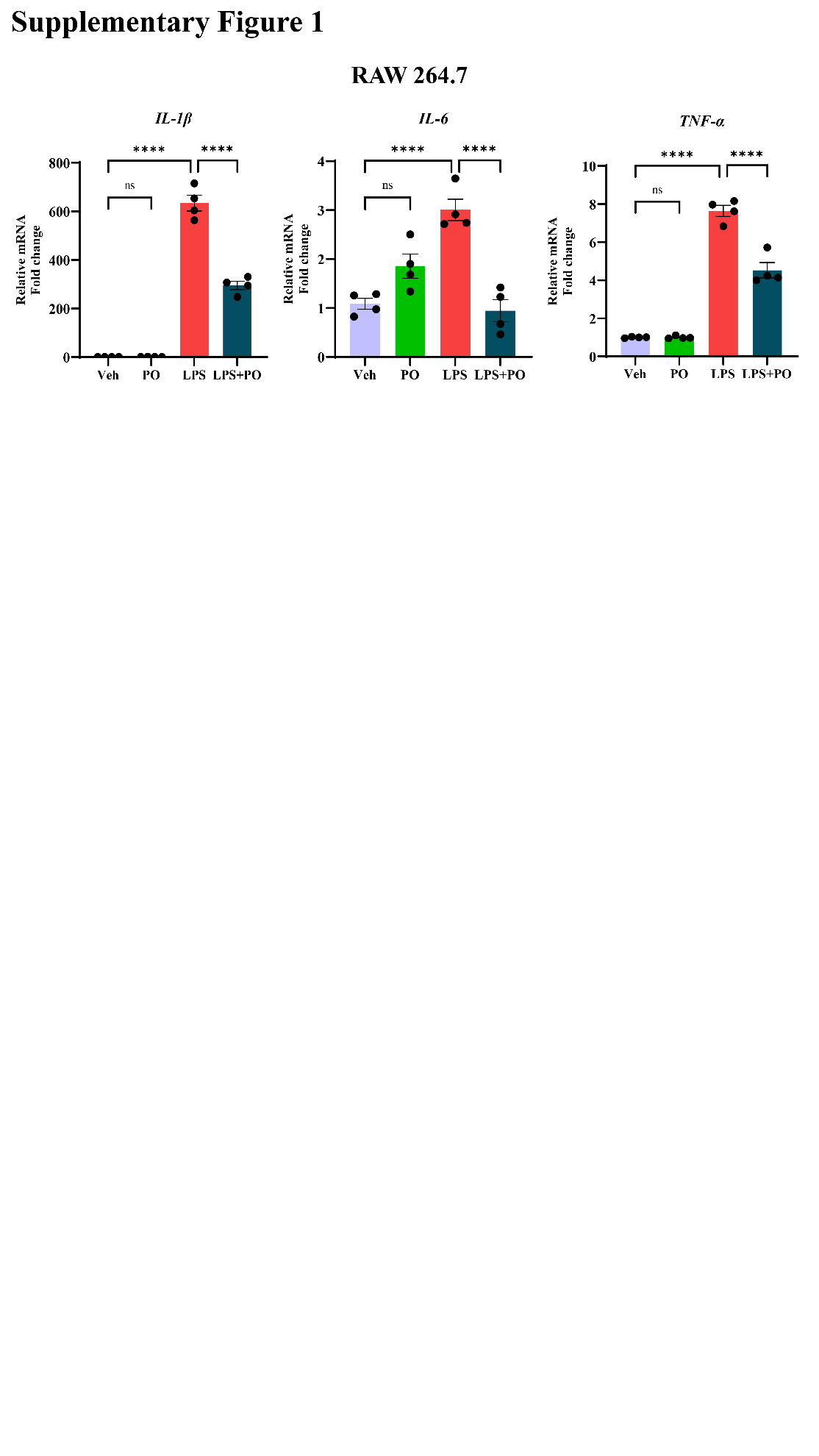
**

**Fig S1. Palmitoleate prevent LPS-induced Inflammation mouse RAW264.7 macrophages.** RAW cells were exposed with LPS (10 ng/ml) for 3 hr to mimic inflammation, *in vitro*. LPS increased the expression of proinflammatory cytokines like *IL-1b, IL-6 and TNFα* mRNA relative to control *36B4* mRNA expression. Pre-treatment of palmitoleate (PO), 200 µM significantly decreased the mRNA levels of proinflammatory cytokines in BMDMs with LPS. These data represent the mean ± SEM for n=4. **** p<0.0001.
